# Visual Representation of Red Wine Color: Methodology, Comparison and Applications

**DOI:** 10.3390/foods12050924

**Published:** 2023-02-22

**Authors:** Shuyue Fan, Caiyun Liu, Yunkui Li, Yu Zhang

**Affiliations:** 1 College of Enology, Northwest A & F University, Yangling 712100, China; 2 Ningxia Helan Mountain’s East Foothill Wine Experiment and Demonstration Station of Northwest A & F University, Yinchuan 750104, China

**Keywords:** color visualization, color characterization, color reproduction, wine fermentation, CIELAB

## Abstract

A visual and easy-to-implement representation approach of red wine color is proposed in this work. The wine color under standard conditions, called feature color, was reproduced in the form of a circular spot. The feature color was further decomposed into two orthogonal aspects, the chromatic and light–dark components, characterized in the form of chromaticity distribution plane and lightness distribution plane, respectively. The color characterization of wine samples showed that this method well represented the color characteristics and can provide intuitive visual perception of wine color, in a way that is more reliable and convenient than the photographic method. The applications for monitoring the color evolution during winery and laboratory fermentation and the age discrimination of 175 commercial red wines suggest that this visual method is effective for color management and control of wine during fermentation and aging. The proposed method is a convenient way to present, store, convey, understand, analyze and compare the color information of wines.

## 1. Introduction

As an intuitive sensory characteristic and important quality indicator of red wine, color is usually considered to be the first sensory attribute experienced by consumers [1], and is a key factor that directly affects consumer acceptance and preference of red wine [2]. Color also provides information about evolution, development and even possible defects of red wine during aging and storage. Therefore, color is a crucial regulating factor for quality control of red wine [3]. Scientifically determining the color of red wine, and conveniently storing, analyzing, comparing and transmitting the color information of red wine, is vital for wine researchers and producers.

The commonly used qualitative assessment of the color of red wine is the sensory description method, which requires the taster to be a professional, and is affected by the condition of the tasters and even the condition of the observation [4]. Needless to say, the color information of red wine carried by sensory description is limited, because the subjective differences of the tasters are not negligible, and the color information is significantly subject to observation conditions such as the illumination and geometric shape of wines in the glass. In addition to being limited to subjective differences and observation conditions, sensory descriptions are less transmissible and can cause misunderstandings among people with different cultural backgrounds and language systems. For example, the transfer of sensory descriptors between different language systems will involve translation and understanding across different cultural backgrounds, which seems likely to cause some difficulties in communicating the particular colors indicated by sensory descriptors. Photography (digital image or computer vision system), another commonly used qualitative recording method of red wine color, seems to compensate for the limitations of sensory description. Photographs (digital vision image) could show a macro-panorama including visual structure, and distributions of color, gloss, shade and translucency of red wine color, to intuitively perceive the color features of red wine [1,5]. Nevertheless, making images still does not seem to be the optimal solution for red wine color information description. High demands on professional photographers, specialized equipment and strict conditions that are not easily accessible to most researchers and producers restrict the application of this method [6].

The above-mentioned two qualitative red wine color description methods represent the red wine color appearance outline and characteristics from the macro- and human-eye-perception perspective, and the quantitative red wine color measurement method conveys more precise numerical and digital information regarding red wine color from an objective perspective. The traditional method for quantitative measurement of wine color is the Glories parameter method [7], which has been widely used because it is simple and easy to implement and can reflect the color characteristics of wine samples to a certain extent [8]. This method employs several parameters, the color hue *H*, the color density *CD* and the color intensity *CI*, to express the color information of red wine by visible absorbance values at several specific wavelengths (420, 520 and 620 nm). Alternatively, a uniform color space CIELAB [9] established by the Commission Internationale de l’Eclairage (CIE) is able to achieve a better consistency between visually perceived color and the color parameters determined by the CIELAB method [10]. Therefore, it has been recommended by the International Vine and Wine Organization (OIV) [11] and widely used in wine color determination. The three-dimensional color space CIELAB with high uniformity is constructed using three parameter axes, a* (red–green color channel), b* (yellow–blue color channel) and L* (lightness), representing different color features. It is easily conceivable and parameters L*, $C_{\mathrm{ab}}^{*}$ (chroma or saturation) and $h_{\mathrm{ab}}^{*}$ (hue angle) are more closely related to the color properties of visual and psychophysical perception [12].

Although the CIELAB color parameters with visual uniformity are easily conceivable, it seems difficult to directly and intuitively present the visible color information behind the numerical parameter values. In order to overcome this difficulty as much as possible, we proposed a visual representation method of red wine color based on CIELAB color space. This method constructs a chromaticity plane and a lightness plane to render the color information of red wine, and can reproduce the feature color of wine. Subsequently, we evaluated the representativeness of this method, and we provide two applications for reference.

## 2. Materials and Methods

### 2.1. Materials for Representation, Comparison and Application

#### 2.1.1. Materials for Representation and Comparison of Red Wine Color

Twelve commercial Cabernet Sauvignon dry red wines from different wineries in 5 provinces in China were used to study the visual representation of red wine color and to undertake the subsequent comparison (Table 1).

In order to preserve and restore the true color of red wine samples as much as possible, a Canon 60D camera, Stone Island ISO wine glass (Shandong Huapeng Glass Co., Ltd., Weihai, China), standard D65 white light illuminant (MASTER TL-D 90 De Luxe 36w/965 6500K, Philips, Wroclaw, Poland), fixed bracket and small studio were used to construct the photographic conditions required to take real photos of 12 red wine samples. 

#### 2.1.2. Materials for Monitoring the Evolution Trajectory of Red Wine Color during Fermentation

Samples of fermentation on a winery scale were taken once a day from a 30,000 L fermentation tank during the commercial Cabernet Sauvignon grapes fermentation process in Ningxia Helan Mountain’s East Foothill Wine Experiment and Demonstration Station of Northwest A&F University, Ningxia Province, China (106°27′ E, 38°47′ N) in October 2021. All samples were filtered through nylon cloth of 200 mesh (mesh size 0.048 mm) after sampling and subsequently frozen at −80 °C.

Samples of fermentation on a laboratory scale were taken once a day from a 5 L glass container during the manual Cabernet Sauvignon grapes fermentation process in a laboratory in October 2021. All samples were centrifuged at 7500× *g* for 15 min (HC-30182; Anhui USTC ZONKIA Scientifc InstrumentsCo., Ltd., Hefei, China) after sampling and subsequently frozen at −80 °C.

#### 2.1.3. Materials for Distinguishing Wine Age

This part of the study involved 175 commercial dry red wines from different wineries in Ningxia Helan Mountain East Foothill area, China, with ages ranging from 1 to 6 (Table 2). Moreover, most of them were monovarietal wines fermented by single grape variety, such as Cabernet Sauvignon, Merlot, Cabernet Gernischt or Marselan; a few of them, though, were multivarietal wines blended by two or three grape varieties.

### 2.2. Measurement of Visible Absorption Spectrum 

Each sample taken from two scales of fermentation was thawed at room temperature and centrifuged at 7500× *g* for 15 min (HC-30182; Anhui USTC ZONKIA Scientifc Instruments Co., Ltd., Hefei, China). Each red wine sample was filtered using 0.45 μm polyether sulfone membranes (Shanyutech, Co., Ltd., Tianjin, China) before measurement [13].

The visible absorption spectrum (400~780 nm, 1 nm interval) of each sample included 12 red wine samples; samples taken from two scales of fermentation and 175 commercial red wine samples were scanned and recorded using a spectrophotometer, Agilent Cary 60 UV-Vis (Agilent Technologies Inc., PaloAlto, CA, USA), with a 1 mm path length quartz cuvette (i-Quip^®^, Aladdin Biochemical Technology Co., Ltd., Shanghai, China). Deionized water was set as the blank reference. Each analysis was performed in triplicate.

### 2.3. Calculation of CIELAB Color Parameters

The CIELAB color parameters (L*, a*, b*, $C_{\mathrm{ab}}^{*}$ and $h_{\mathrm{ab}}^{*}$) of each wine sample were calculated using absorbance values at four wavelengths (450 nm, 520 nm, 570 nm and 630 nm) according to the simplified method [14] with minor modification. Moreover, calculation followed the conditions of a 10° observer viewing angle with standard D65 illuminant.

Parameters L*, a* and b* were calculated according to the Formulae (1)–(8):

(1) $$T_{i}=10^{-A_{i}}\;(i=450,\;520,\;570,\;630)$$

(2) $$X=19.717T_{450}+1.884T_{520}+42.539T_{570}+32.474T_{630}-1.841$$

(3) $$Y=7.950\;T_{450}+34.764\;T_{520}+42.736\;T_{570}+15.759\;T_{630}-\;1.180$$

(4) $$Z=103.518\;T_{450}+4.190\;T_{520}+0.251\;T_{570}-\;1.831\;T_{630}+0.818\;$$

(5) $$L^{*}=116\;f\left(\frac{Y}{Y_{n}}\right)-16$$

(6) $$a^{*}=500\;\left(f\left(\frac{X}{X_{n}}\right)-f\left(\frac{Y}{Y_{n}}\right)\right)$$

(7) $$b^{*}=200\;\left(f\left(\frac{Y}{Y_{n}}\right)-f\left(\frac{Z}{Z_{n}}\right)\right)$$

(8) $$f\left(I\right)=\left\{\begin{array}{c} and\sqrt[{3}]{I}\;\;\;\;\;\;\;\;\;\;\;\;\;\;\;\;\;\;\;\;(I>0.0089)\\ and\frac{841}{108}I+\frac{4}{29}\;\;\;\;\;\;\;\;\left(I\leq 0.0089\right) \end{array}\right.$$

where *A* is the absorbance; *T* is the transmittance; *X*, *Y* and *Z* stand for the tristimulus values of the sample; and *X_n_*, *Y_n_* and *Z_n_* denote the tristimulus values of standard D65 illuminant, assigned as 94.825, 100.000 and 107.381, respectively.

Chroma $C_{\mathrm{ab}}^{*}$ and hue $h_{\mathrm{ab}}^{*}$ were calculated according to L*, a* and b*:

(9) $$C_{\mathrm{ab}}^{*}=\sqrt{a^{*2}+b^{*2}}$$

(10) $$h_{\mathrm{ab}}^{*}=\arctan\frac{b^{*}}{a^{*}}$$

Moreover, color difference $\Delta E_{\mathrm{ab}}^{*}$ was calculated according to CIEDE2000 color-difference formula [15]:

(11) $$\begin{array}{c} \Delta E_{00}^{12}=\Delta E_{00}\left(L_{1}^{*},a_{1}^{*},b_{1}^{*},L_{2}^{*},a_{2}^{*},b_{2}^{*}\right)\\ =\sqrt{{\left(\frac{\Delta L^{\prime }}{K_{L}S_{L}}\right)}^{2}+{\left(\frac{\Delta C^{\prime }}{K_{C}S_{C}}\right)}^{2}+{\left(\frac{\Delta H^{\prime }}{K_{H}S_{H}}\right)}^{2}+R_{T}\left(\frac{\Delta C^{\prime }}{K_{C}S_{C}}\right)\left(\frac{\Delta H^{\prime }}{K_{H}S_{H}}\right)} \end{array}$$

Color difference $\Delta E_{00}^{12}$ was calculated under the following reference conditions:

$$K_{L}=K_{C}=K_{H}=1$$

$\Delta E_{00}^{12}$ indicates the color difference between two sample points (1 and 2) in the CIELAB color space.

### 2.4. Visual Representation

The visual representation of red wine color is realized based on the three-dimensional CIELAB color space. Its fundamental logic is to decompose the three-dimensional color space into two dimensions and one dimension, so as to achieve the purpose of visually representing the color information contained in the three-dimensional color space. When L* is specified, a* and b* form a two-dimensional chromaticity plane with a certain lightness. Correspondingly, when a* and b* are both set to be 0, L* represents a one-dimensional lightness coordinate axis without chromaticity information (a* = 0, b* = 0). Dimensionality reduction means disassembly of the color space. Three dimensions are reduced to two dimensions and one dimension, implying that the color space is disassembled into a chromaticity plane and lightness axis. 

Considering the average lightness (L* = 69.32) and value ranges of L* (41.59–91.20), a* (9.20–50.80) and b* (−4.57–32.25) of the 403 red wines involved in research in our laboratory (Table S1), a two-dimensional a* − b* (a* = 0~60, b* = −20~40) chromaticity plane with a certain lightness (L* = 70) was constructed, which covers almost all possible chromaticities of red wines; although young blue-toned wines with very small b* values were not involved in this study, the chromaticity plane (b* = −20~40) fully takes into account the possible existence of such wines. Meanwhile, a one-dimensional lightness axis (L* = 40~100) that can cover almost all possible lightness values of red wines was established. Then, points in the CIELAB color space that correspond the color of a series of red wine samples can be projected on the chromaticity plane in order to present their chromatic properties conveniently. Thus, this plane is called the chromaticity distribution plane, on which the chromatic performance of a wine or the chromaticity distribution of a series of wine samples can be exhibited and compared intuitively. Similarly, points can be projected on the plane extended by the lightness axis, which is called a lightness distribution plane. Therefore, the lightness degree without any chromaticity information about a wine or the lightness distribution of a series of wine samples can be demonstrated and compared directly. 

By means of these two planes, the color information of wine can be decomposed into two orthogonal factors, which is helpful both for revealing the formation of a wine color and for comparing the color difference of various wines. In addition, the reproduction of the overall color of wine is likewise an important concern. Accordingly, a circular spot was constructed and colored in consideration of all the contributions of L*, a* and b*. It is called the feature color, which represents the color of wine body at a specific liquid thickness (e.g., 1 mm) in natural observation conditions (standard D65 illuminant and 10° viewing angle). 

It should be emphasized that the construction of the visual representation of red wine color is based on the visible absorbance spectrum at a specific optical path. With consideration of the absorbance value range of red wine and Beer–Lambert law, 1 mm optical path is recommended for the measuring of the visible absorbance spectrum to obtain the optimal response of the UV-Vis spectrophotometer. Moreover, comparisons between different studies using the suggested optical path will be meaningful.

### 2.5. Statistical Analysis

Excel 2016 (Microsoft, Redmond, Washington, DC, USA) was used for data calculations and statistics; SPSS Statistics 20 (IBM, New York, NY, USA) was used to complete the main data analysis; and Photoshop CS6 (Adobe, San Jose, CA, USA) was used to draw the artworks.

## 3. Results and Discussion

### 3.1. Visual Representation of Red Wine Color 

**Figure 1:**
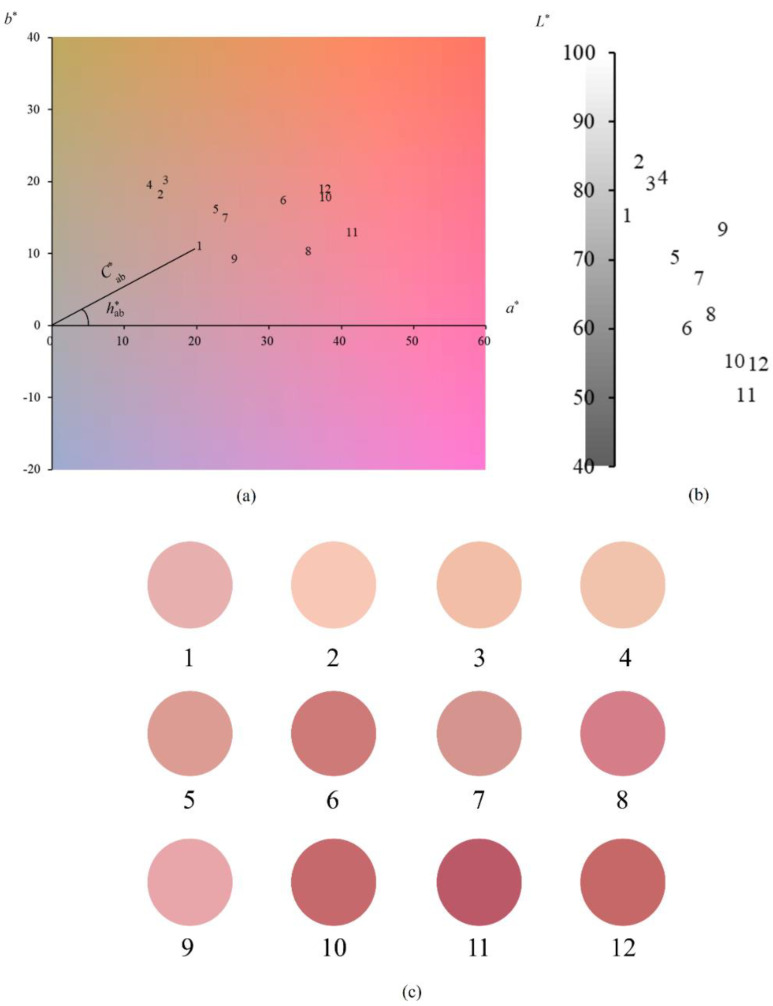
Chromaticity distribution plane (**a**), lightness distribution plane (**b**) and feature colors (**c**) of 12 red wine samples. The feature color contains all the contributions of L*, a* and b*.

Based on the aforementioned methods, 12 wine samples were used as examples to further demonstrate and illustrate the visual representation of red wine color. First, CIELAB color parameters (Table 1) of the wine samples were calculated using the visible absorbance spectra (Figure S1). Next, each wine sample point in the color space was projected to obtain the chromaticity distribution plane (Figure 1a) and lightness distribution plane (Figure 1b). Finally, the feature colors that combine the contributions of L*, a* and b* were rendered (Figure 1c).

In CIELAB system, L*, a* and b* construct a three-dimensional color space, of which the parameter L* expresses the lightness information. For the D65 illuminant, the values of L* range from 0 (black) to 100 (white) [16]. The L* value’s range for the 12 red wine samples is 50.46 (wine sample No. 11) to 84.19 (No. 2); the L* values of different samples vary significantly from each other (Table 1). Corresponding to the lightness distribution plane (Figure 1b), the overall lightness distribution of the 12 wine samples and the lightness feature of each wine sample are clear at a glance.

The a* − b* plane reserves the chromatic information. The parameter a* represents the red (+)-green (−) color channel. The parameter b* denotes the yellow (+)-blue (−) color channel [17]. The 12 red wine samples have a* values between 13.47 (No. 4) and 41.46 (No. 11), of which there are significant differences between the wine samples except for No. 10 and No. 12. The b* values range from 7.05 (No. 9) to 18.06 (No. 3); the b* values of the different samples are significantly different from each other (Table 1). 

The parameter $C_{\mathrm{ab}}^{*}$, also called chroma, is the color saturation varying from brilliant to bright (from dull to vivid). $C_{\mathrm{ab}}^{*}$ is considered as the quantitative property of color, allowing one to assess the degree of difference related to a gray color exhibiting the same lightness for each hue [18]. The larger the value of $C_{\mathrm{ab}}^{*}$, the greater the difference between the hue and the gray at the same level of lightness. In other words, a larger chroma implies that the color is more concentrated, vivid and intense and the saturation of the color is higher. On the contrary, the smaller the difference between the hue and the gray at the same level of lightness, the more the gray contributes to the color, resulting in a duller color and lower saturation. The $C_{\mathrm{ab}}^{*}$ of the 12 wine samples ranges from 22.00 (No. 4) to 42.84 (No. 11); there are significant differences among the wine samples except for No. 2 and No. 4 (as shown in Table 1).

The parameter $h_{\mathrm{ab}}^{*}$, also called hue angle, is regarded as the qualitative property of color [16], and is used to characterize the total and general tendency of color, such as reddish ($h_{\mathrm{ab}}^{*}$ = 0°) or yellowish ($h_{\mathrm{ab}}^{*}$ = 90°). The $h_{\mathrm{ab}}^{*}$ of the 12 wine samples ranges from 13.03 (No. 8) to 52.20 (No. 4); the $h_{\mathrm{ab}}^{*}$ values of the different samples are significantly different from each other (Table 1).

The above parameters a*, b*, $C_{\mathrm{ab}}^{*}$ and $h_{\mathrm{ab}}^{*}$ and their relationship with each other as well as the chromaticity features of wine samples referred to by these parameters are visually and intuitively displayed on the chromaticity distribution plane (Figure 1a). The color behind each wine sample number is the color information of the wine sample that is contributed to by the a* and b* parameters. The straight-line distance between the wine sample point and the origin reveals the saturation $C_{\mathrm{ab}}^{*}$ of the wine color, and the angle between the straight line and the a*-axis means the hue angle $h_{\mathrm{ab}}^{*}$ of the wine color. Therefore, the chromaticity distribution plane completely presents the color information of each wine, including comprehensive color and the contribution of different color parameters. In addition, such as for wine sample eleven with the largest a* and the largest $C_{\mathrm{ab}}^{*}$, and for wine sample four with the smallest a* and the largest $h_{\mathrm{ab}}^{*}$, their chromaticity features differ significantly from each other, as shown in Figure 1a. The chromaticity distribution plane intuitively displays the wine color information difference between wine samples. The difference of comprehensive colors, each color’s parameters and various color properties are all visually revealed by the plane, although the difference is subtle. 

The chromaticity distribution plane and the lightness distribution plane are helpful to analyze and compare the chromaticity information and lightness information of the wine samples in detail. However, a sample’s feature color in a circular spot can show a more macroscopic color visual perception from an overall perspective (Figure 1c). Thus, the combination of chromaticity distribution plane, the lightness distribution plane and the feature color can completely and visibly present color information of wine samples. Additionally, the combination is a useful and convenient tool to study wine color rather than solely relying on the simple CIELAB color parameters (Table 1) alone.

It seemed relatively difficult for the human eye to distinguish the feature colors of three and four, as well as ten and twelve, so the color difference $\Delta E_{00}$ of each pair was calculated. The color difference between 3 and 4 is $\Delta E_{00}^{34}$ = 1.73, and between 10 and 12 it is $\Delta E_{00}^{1012}$ = 0.87. The $\Delta E_{00}$ refers to the total difference in degree of color between samples. The larger the value, the more significant the difference is. Moreover, the $\Delta E_{00}^{34}$ = 1.73 > $\Delta E_{00}^{1012}$ = 0.87; correspondingly, the visually perceived color difference of the feature colors of 3 and 4 is actually more significant than it is between ten and twelve, which gives the human eye an obviously stronger sense of color difference. Specific to the feature colors of three and four, visually, the color of three is more bright, vivid and deep, while the feature color of four seems to look relatively gloomy and pale. Therefore, the feature color could visually and intuitively present the macroscopic color profile of wine samples and the color differences between them, and the visually perceived color difference of feature colors is also consistent with the calculated color difference $\Delta E_{00}$. In addition, the differences between the feature colors of the remaining wine samples could be visually perceived and easily identified. 

Color is a visual art in essence, and the traditional CIELAB color parameter is the digitization of color information to store, transmit, analyze and compare color information conveniently. A previous study decomposed the three-dimensional CIELAB color space into two dimensions (a*b*diagram) and one dimension (lightness value), in order to intuitively present the differences in parameter values [19]. However, such colourless presentation made the visual perception of the color referred to by the parameter seem distant and fuzzy. The visual representation proposed in this study connects the parameters and the color information perceivable by human eyes, and intuitively presents the chromaticity information and lightness information indicated by the parameters, breaking the barrier between digital quantification and visual perception.

### 3.2. Comparison

**Figure 2:**
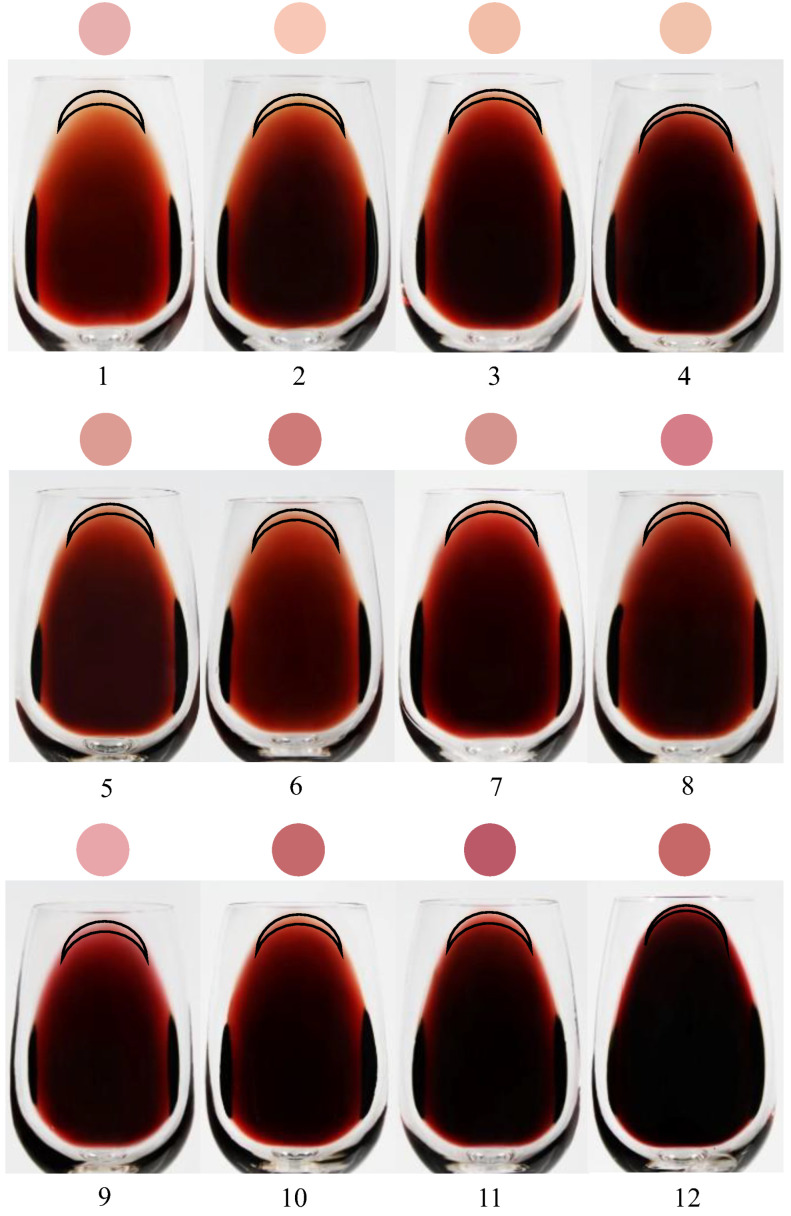
Comparison between the feature color and realistic photo of each wine sample.

From the mechanism of visual representation of red wine color, the feature color could represent the color of a uniform red wine liquid at a depth of 1 mm on a white background. In order to evaluate the representativeness of the feature color, i.e., whether the feature color is able to represent the color of real red wine as visually perceived by the human eye through the wine glass, we compared the feature color and the real photo of each wine sample. The feature color represented by a circular spot, and the real uniform color inside the crescent circle at the rim of each red wine in the wine glass recorded by digital photography, are presented in Figure 2. 

As can be seen from the color at the edge of the thin wine liquid layer in the real photos of the red wine samples, wine samples 9, 10, 11 and 12 have a relatively strong red hue. Correspondingly, each feature color also shows a relatively strong red hue to a certain extent. However, wine samples one, two, three and four exhibit a pronounced bright and pale yellow hue. Correspondingly, each feature color also shows this characteristic. Wine samples five, six and seven show brick red and tile red around the edges, and their feature colors also reflect the main hue. The feature color of wine sample eight does not fully express the brick red around its edge, but instead has a more purplish red hue, which seems to be tolerable. On one hand, from the perspective of the real photo, there seems to be a subtle distinction that is hard to distinguish by the human eye between the wine samples five, six, seven and eight. However, on the other hand, from the perspective of the feature color, we could easily distinguish and identify color differences between the four wine samples and justifiably conclude that, of samples five, six, seven and eight, wine sample eight presents the best color, with a significant red hue, and wine sample six shows obvious deeper color perception. Therefore, compared with the color information recorded by photography, the feature color of wine could distinguish the more subtle color difference between wine samples while presenting the clear macroscopic color. This can also be further explained by the metameric properties of red wine explored in one study [20]. This means that visually the same colors actually have different spectrums, so that spectral data are more reliable in reproducing and distinguishing these same colors. In other words, the feature color of wine essentially based on spectral data could reliably distinguish subtle color differences and even visually the same colors.

In short, the feature color could relatively closely represent the general tendency of the real hue of the wine edge, but does not seem to be a good representative of the other two key properties, chroma and lightness. This may be attributed to the effect of background color differences, since even though the real photos were taken under standard conditions, the background appears light gray, which will significantly affect perception and discrimination of the two key color properties related to gray degree: chroma and lightness. Moreover, the feature colors with a pure white background could avoid the potential influence of the background. However, it must be admitted that feature colors cannot express the layered and varied appearance of red wines under usual observing conditions (e.g., in a wine glass) [21], because red wine is semitransparent liquid, whose color is significantly affected by the thickness (depth) and vessel [22]. Although we compared the feature color with the color of the red wine rim to exclude the influence of thickness as much as possible, the effect of texture and transparency of the liquid medium is still relatively difficult to control.

Visual color measurement and photography are traditional methods of qualitative measurement of red wine color, which can perceive and represent the layered and varied appearance of red wines, but they have strict requirements on measurement conditions, equipment and operators, and the color of red wine under non-standard conditions is inaccurate and diverse [4]. In other words, red wine color is a complex and comprehensive sensation based on multiple factors such as the illuminant, space, position, subjective feelings and preferences, which may indicate that color is not only a property of the wine sample itself, but also a combined response to complex factors [23]. From this point of view, the normative instrumental color measurement eliminates the interference of complex factors and returns to the color itself, that is, the response of the red wine sample to visible light, so it provides a more simple and reliable measurement of color. Based on precise instrumental color measurement, feature color represented the instrumental color of red wine and proposed a novel color reproduction scheme for red wine. Although the instrumental color of red wine is somewhat different from the visual color [24], theoretically, the instrumental color is undoubtedly more objective, true, accurate and standard, because the instrument is superior to the human eye’s ability to discriminate colors. Moreover, compared to the reproduction of the color of red wine by photography [4], synthesizing liquid [2] or digital images [23], the feature color provides a more accurate and convenient solution for visually representing, reproducing and retracing the color of wine.

### 3.3. Application

On the basis of the visual representation of red wine color proposed in this work, several attempts at color management can be turned into reality. We have tried this in several respects, including monitoring color evolution during red wine fermentation, distinction of red wine vintages, and classification and grading of red wine. The first two points will be applied in the following, while the last one will be systematically presented in another paper. 

#### 3.3.1. Monitoring Red Wine Color Evolution Track during Fermentation

**Figure 3:**
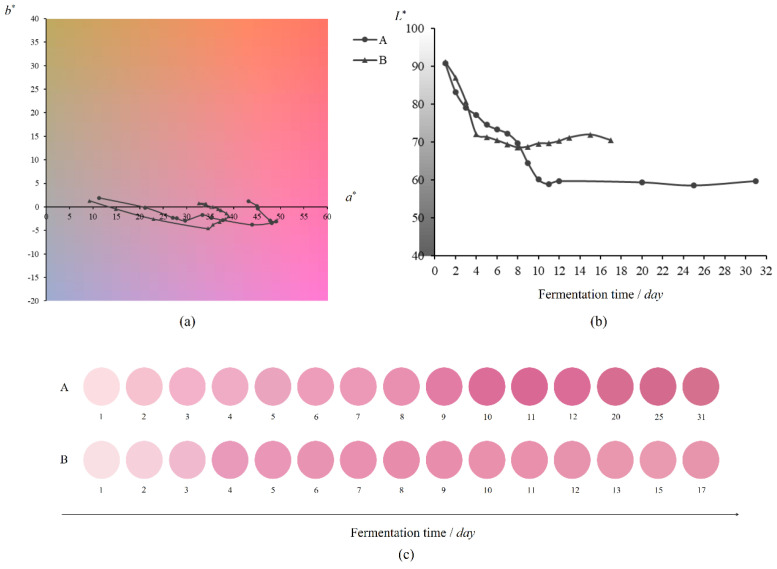
Chromaticity (**a**), lightness (**b**) and feature color (**c**) evolution track of red wine color during fermentation. (**A**): Fermentation on a winery scale; (**B**): Fermentation on a laboratory scale.

Fermentation on a winery scale and a laboratory scale were conducted. During these fermentations, the formation and evolution features of color were monitored. The result is shown in Figure 3. It is evident that the color evolution track of red wines followed a similar trend during both winery and laboratory scales of fermentation, even though the fermentation times on the two scales were not completely synchronized. Focusing on the chromaticity evolution track of red wine color during the fermentation (Figure 3a), we can intuitively find that in the early and middle phases of fermentation, the color parameter a* (indicating the red attribute) continued to increase, and the color parameter b* (which indicates the yellow attribute) decreased continuously at the same time in the winery scale, but in the laboratory scale, the color parameter b* first decreased continuously, and then increased slightly. At the late phase of fermentation, the color parameter a* began to gradually decrease, and simultaneously, the color parameter b* began to increase slowly in the winery scale, and continued to increase slightly in the laboratory scale. Previous studies have reported a trend similar to that found in this study, that is, that in the late phase of alcoholic fermentation, the parameter a* decreased slowly [25] and b* increased gradually [26], leading to an inflection point of chromaticity change. The inflection point discovered in this study is a crucial way of controlling red wine color quality during fermentation, which is rarely reported in other studies. Moreover, the material basis and mechanism behind this change, e.g., the matrix effect on the formation of sensory characteristics [27], deserve further exploration.

In addition to the changes in the value of color parameters a* and b*, we can also perceive the chromaticity evolution through the chromaticity plane. As the fermentation phase advances, the color changes from an initially dull greenish yellow with slightly pinkish hue to the most vivid purplish red in the period, and finally a vivid cochineal. It can be seen from Figure 3b that during the fermentation of the two scales, the lightness evolution track of the red wine color continued to decline from the same initial lightness to different levels of lightness, and finally remained steady.

From above, we could reasonably speculate that the evolutions of chromaticity and lightness are closely related to the dissolution of substances, such as anthocyanins and polyphenols, during the early and middle phases of fermentation [28,29,30]. Meanwhile, in these phases, b* continuously declined, which indicates that the browning caused by oxidation is extremely slight. However, during the last phase of fermentation, b* began to slowly increase, which implies that oxidation might become relatively strong because of the reduced CO_2_ yield [31]. Moreover, in the whole fermentation, the formation of anthocyanin derivatives and the interaction between phenolic species undoubtedly contributed to the evolution of the red wine color [32,33,34]. Therefore, monitoring the color evolution during red wine fermentation can enable understanding of the changes in color-related substances from the side.

The feature color of red wine comprehensively considers the chromaticity and lightness of the red wine color, and as the final complete macro-color perceived by the human eye, presents the evolution track in the fermentation of two scales (Figure 3c). Apparently, the change in feature color is significant. As the fermentation progresses to the middle phase, the purplish red hue became enhanced, the saturation (vividness) became higher, and the lightness decreased, which is the dominant trend. However, in the late phase, this trend did not continue: the hue began to contain a little yellow, and the saturation also decreased slightly. In other words, the relatively vivid purplish red was starting to convert into a vivid cochineal. It is worth mentioning that the fermentation process and duration of the two scales are not synchronized. Ultimately, the chromaticity and lightness of the red wine color reaches different levels in the two scales, resulting in different feature colors, which can be attributed to the differences of raw grape materials, fermentation containers and winemaking techniques [35].

All in all, it is efficient and intuitive to use the visual representation method to trace the color evolution track of the fermentation. Undoubtedly, the monitoring of color changes can be extended to the stable, mature, aging and storage stages of the red wine after fermentation, so as to better understand and grasp the color change trajectory of the whole life cycle of red wine. 

#### 3.3.2. Distinction of Red Wine Ages

**Figure 4:**
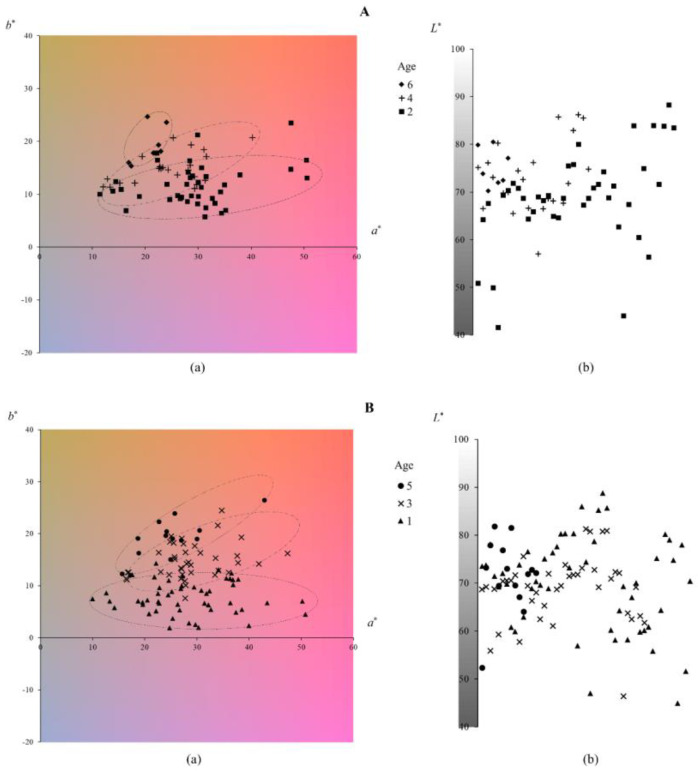
Chromaticity (**a**) and lightness (**b**) distinction of 70 red wine samples in group A (**A**) and 105 red wine samples in group B (**B**) of different ages. Dotted ellipses on the chromaticity plane mark the concentrated distribution of wine samples of each age.

The 175 red wine samples were divided into two groups (Table 2) based on their ages in order to set the same age interval for each group of wine samples to more clearly show the relationship between age and color information. Group A contained 70 samples of the ages 6(7), 4(23) and 2(40), and group B contained 105 samples of the ages 5(14), 3(40) and 1(51). Each group is displayed with the visual representation to distinguish the age difference of the wine samples in the group (Figure 4).

It can be seen from the chromaticity distribution plane (Figure 4(Aa,Ba)) that although different wine samples are scattered, the wine samples of the same age still show a certain degree of concentrated distribution marked by the dotted ellipse. Moreover, each dotted ellipse marks the distribution area of 90% or more of wine samples of each wine age. Paying attention to these concentrated distribution areas, we can see that wines of different ages have a certain degree of distinction. Compared with other chromaticity parameters (a*, b* and $C_{\mathrm{ab}}^{*}$), this distinction is particularly noticeable in the difference of hue $h_{\mathrm{ab}}^{*}$. Younger wine samples are smaller in hue $h_{\mathrm{ab}}^{*}$, appearing more purple and reddish, and conversely, older wine samples have a larger hue $h_{\mathrm{ab}}^{*}$, appearing more brick red or even yellower. It is undeniable that the hue $h_{\mathrm{ab}}^{*}$ of red wine samples does have a distribution rule related to the age, and a Pearson correlation analysis manifested that the correlation coefficient between the hue value and the wine age is 0.707 (*p* < 0.01). In contrast, hue $h_{\mathrm{ab}}^{*}$ could distinguish age differences to some extent. Nevertheless, wine samples of different ages cannot be completely distinguished, and cross-mixing distribution of sporadic wine samples is inevitable. It is well known that storage and aging lead to yellowing of the color of red wines over time [36], especially when measuring the color of the same red wine at different times [37,38], because other factors affecting the color of red wines are controlled and the aging is amplified. However, the relationship between age and color was weakened when comparing the color of different red wines of various ages, as presented in this study, because other factors affecting the color of red wines, such as grapes, winemaking techniques, additives and storage conditions, etc., cannot be controlled and ignored [39,40]. Nonetheless, this study confirms that even though the color of red wine is influenced by many complex factors, the effect of age cannot be underestimated, especially when the ages vary widely, such as an age difference of two years or more. 

The lightness values of the wine samples in groups A and B are mostly distributed at L* = 60~80, and concentrated around L* = 70, showing a slightly bright appearance (Figure 4(Ab,Bb)). Regrettably, there does not seem to be a sufficient difference in lightness of various ages to distinguish the age. Although there have been studies reporting that the same red wine increases in lightness L* with aging [37], few studies have reported the differences in lightness L* between different red wines of various ages.

In fact, the application of the visual representation method is not only manifested in the above-mentioned aspects. In any scientific research and practice process that may involve the color representation of red wine, this method can provide potential applications. 

## 4. Conclusions

A visual representation method was proposed in this work to visualize and reproduce red wine color based on CIELAB color space. The practicability and innovation of this method were further demonstrated adequately in three respects. Conclusions were drawn as follows:

1. The visual representation method of red wine color is a useful and convenient approach to present, store, convey, understand, analyze and compare the color information of red wine(s), which could provide intuitive visual perception of red wine color rather than purely digitalized CIELAB color parameters.
2. The visual representation method of red wine color could represent and reproduce the fundamental color characteristic of real red wine, such as the overall color tendency, although the approach could not perfectly display the translucent and layered appearance of real wine. In addition, the feature color is a more reliable and simpler way to reproduce the red wine color and identify nuances of the color than photography.
3. The feasibility of this visual representation method in practice was certified in two applications, i.e., monitoring the color evolution during wine fermentation, and the discrimination of wine age. Its good behavior in the two applications of this method suggests that this visual method of color is an effective tool and may be extended to any situation including wine color or even other beverage systems.

To sum up, color itself is an important aspect of red wine quality. Meanwhile, color suggests some information, such as vintage, variety, possible defects, etc., and is related to many important components. Therefore, the visual representation method provides a new approach and a useful tool to further excavate red wine color information and displays expansive potential in the field of research involving red wine color.

## Figures and Tables

**Table 1:** CIELAB color parameters of red wine samples used for representation and comparison.

Number	Age	Origin	CIELAB Color Parameters				
			***L*** *	***a*** *	***b*** *	$C_{\mathrm{ab}}^{*}$	$h_{\mathrm{ab}}^{*}\;/{}^{\circ}$
1	5	Sichuan	76.43 ± 0.14 ^d^	20.37 ± 0.04 ^h^	8.89 ± 0.03 ^j^	22.23 ± 0.04 ^j^	23.58 ± 0.07 ^g^
2	7	Ningxia	84.19 ± 0.08 ^a^	15.06 ± 0.02 ^j^	16.06 ± 0.07 ^d^	22.02 ± 0.04 ^j^	46.80 ± 0.14 ^c^
3	8	Ningxia	81.11 ± 0.31 ^c^	15.74 ± 0.11 ^i^	18.06 ± 0.11 ^a^	23.96 ± 0.03 ^i^	48.90 ± 0.36 ^b^
4	8	Shanxi	81.99 ± 0.25 ^b^	13.47 ± 0.06 ^k^	17.39 ± 0.06 ^b^	22.00 ± 0.01 ^j^	52.20 ± 0.22 ^a^
5	4	Xinjiang	70.38 ± 0.11 ^f^	22.69 ± 0.06 ^g^	13.99 ± 0.07 ^g^	26.69 ± 0.09 ^g^	31.67 ± 0.06 ^d^
6	3	Neimeng	60.09 ± 0.05 ^i^	31.95 ± 0.02 ^d^	15.27 ± 0.06 ^f^	35.41 ± 0.03 ^e^	25.55 ± 0.10 ^f^
7	5	Ningxia	67.37 ± 0.08 ^g^	23.92 ± 0.05 ^f^	12.73 ± 0.04 ^h^	27.10 ± 0.06 ^f^	28.02 ± 0.06 ^e^
8	2	Ningxia	62.12 ± 0.43 ^h^	35.41 ± 0.10 ^c^	8.19 ± 0.24 ^k^	36.34 ± 0.08 ^d^	13.03 ± 0.39 ^k^
9	5	Sichuan	74.44 ± 0.38 ^e^	25.24 ± 0.21 ^e^	7.05 ± 0.08 ^l^	26.21 ± 0.18 ^h^	15.60 ± 0.28 ^i^
10	3	Neimeng	55.38 ± 0.03 ^j^	37.75 ± 0.07 ^b^	15.63 ± 0.11 ^e^	40.86 ± 0.02 ^c^	22.49 ± 0.18 ^h^
11	4	Ningxia	50.46 ± 0.17 ^k^	41.46 ± 0.11 ^a^	10.78 ± 0.04 ^i^	42.84 ± 0.10 ^a^	14.57 ± 0.09 ^j^
12	3	Xinjiang	54.89 ± 0.04 ^j^	37.71 ± 0.19 ^b^	16.83 ± 0.05 ^c^	41.29 ± 0.17 ^b^	24.00 ± 0.14 ^g^

Data are presented as mean ± standard deviation (*n* = 3). Different letters within a column indicate significant differences at the *p* < 0.05 level by Tukey’s HSD multiple comparisons.

**Table 2:** Age and number of 175 red wine samples used for distinguishing vintage.

Group	Age	N	Sum
A	6	7	70
	4	23	
	2	40	
B	5	14	105
	3	40	
	1	51	

## Data Availability

The data that support the findings of this study are available from the corresponding author upon reasonable request.

## Supplementary Materials

The following supporting information can be downloaded at: https://www.mdpi.com/article/10.3390/foods12050924/s1, Figure S1: Absorption curves of 12 red wine samples in the visible wavelength range (400–780 nm); Table S1: Descriptive statistics of *L**, *a** and *b** of 403 red wine samples.
