# Supplementary material for: Visual Representation of Red Wine Color: Methodology, Comparison and Applications
Source: Foods. 2023 Feb 22;12(5):924. doi: 10.3390/foods12050924 (PMC10000885; doi:10.3390/foods12050924)
Supplement: Supplementary file 1 [file foods-12-00924-s001.zip › foods-2128733-supplementary.pdf]

Supplementary materials

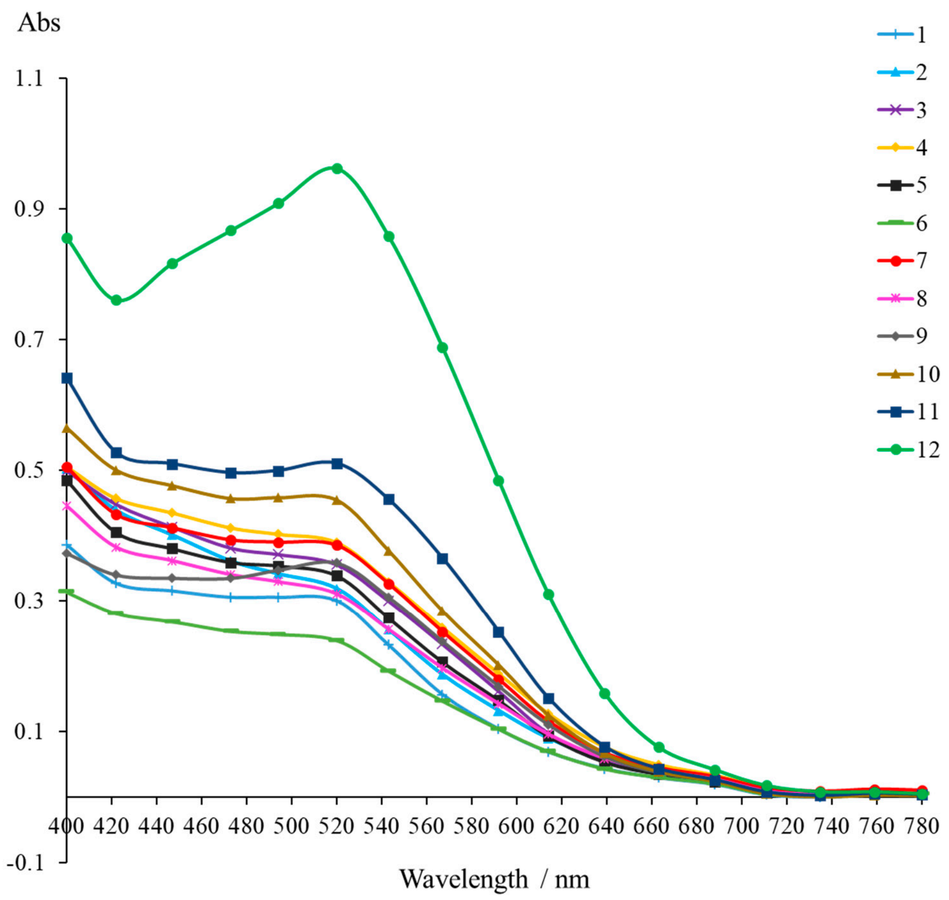

Figure S1. Absorption curves of 12 red wine samples in the visible wavelength range (400–780 nm).

Table S1. Descriptive statistics of L^*, a^* and b^* of 403 red wine samples.

	N	**Range**	**Min**	**Max**	**Mean**	**Standard deviation**
L^*	403	49.61	41.59	91.20	69.32	8.20
a^*	403	41.60	9.20	50.80	29.31	8.50
b^*	403	36.82	-4.57	32.25	11.19	7.18
